# PAX2 is activated by estradiol in breast cancer cells of the luminal subgroup selectively, to confer a low invasive phenotype

**DOI:** 10.1186/1476-4598-10-148

**Published:** 2011-12-14

**Authors:** David Beauchemin, Catherine Lacombe, Céline Van Themsche

**Affiliations:** 1 Research Group in Molecular Oncology and Endocrinology, Department of Chemistry and Biology, Université du Québec à Trois-Rivières, Trois-Rivières, Québec, G9A 5H7 Canada

**Keywords:** PAX2, estradiol, breast cancer, invasion, ERBB2, estrogen receptor alpha, luminal, MCF-7

## Abstract

**Background:**

Metastasis is the leading cause of death among breast cancer patients. Identifying key cellular factors controlling invasion and metastasis of breast cancer cells should pave the way to new therapeutic strategies efficiently interfering with the metastatic process. PAX2 (paired box 2) transcription factor is expressed by breast cancer cells *in vivo *and recently, it was shown to negatively regulate the expression of ERBB2 (erythroblastic leukemia viral oncogene homolog 2, HER-2/neu), a well-documented pro-invasive and pro-metastastic gene, in luminal/ERalpha-positive (ERα+) breast cancer cells. The objective of the present study was to investigate a putative role for PAX2 in the control of luminal breast cancer cells invasion, and to begin to characterize its regulation.

**Results:**

PAX2 activity was higher in cell lines from luminal compared to non-luminal subtype, and activation of PAX2 by estradiol was selectively achieved in breast cancer cell lines of the luminal subtype. This process was blocked by ICI 182780 and could be antagonized by IGF-1. Knockdown of PAX2 in luminal MCF-7 cells completely abrogated estradiol-induced downregulation of ERBB2 and decrease of cell invasion, whereas overexpression of PAX2 in these cells enhanced estradiol effects on ERBB2 levels and cell invasion.

**Conclusions:**

The study demonstrates that PAX2 activation by estradiol is selectively achieved in breast cancer cells of the luminal subtype, via ERα, and identifies IGF-1 as a negative regulator of PAX2 activity in these cells. Further, it reveals a new role for PAX2 in the maintenance of a low invasive behavior in luminal breast cancer cells upon exposure to estradiol, and shows that overexpression and activation of PAX2 in these cells is sufficient to reduce their invasive ability.

## Background

The heterogeneous nature of breast cancer is well established. Based on their expression profile, breast tumours are classified in five molecular subgroups (luminal A and B, basal, ERBB2- overexpressing, and normal-like) [1-3]; each is associated with distinct histological markers and clinical parameters. Only tumours of the luminal subgroups express the receptor alpha to estrogen (ERα); however, luminal A tumours express higher levels of ERα than luminal B tumours [1-3] and they are associated with less aggressive metastatic disease and longer disease-free survival [2]. In accordance, they express low levels of ERBB2 (erythroblastic leukemia viral oncogene homolog 2, HER-2/neu) *in vivo *[1], the expression and activity of which confer invasive and metastatic ability to breast cancer cells [4-7]. *In vitro*, ERα+ lines such as MCF-7 and ZR-75-1, which were derived from ERα+/luminal A tumours, retain a molecular profile characteristic of luminal A tumours, including low expression of ERBB2 [8]; they also display poor invasive and metastatic ability [3,8,9]. These cell lines thus represent a good model to investigate the cellular and molecular mechanisms underlying the poor invasive and metastatic phenotype of luminal A tumours.

PAX2 (paired box 2) is a member of the PAX family of transcription factors, best known for their role in terminal differentiation during organogenesis, at the time of embryo development [10]. PAX2 is involved in kidney and breast development [11,12], and although its expression was initially thought to be silenced at the end of the process [13], expression of PAX2 has been found in adult, differentiated breast cells [14]. PAX2 expression was also detected in breast tumours: although a relatively small number of samples have been analyzed to date, PAX2 expression can equally be found in different types of tumours [12,14-16]. On the other hand, a differential regulation of PAX2 activation has been evidenced between breast tumour subtypes: nuclear localization of PAX2 is frequent in luminal tumours and infrequent in non-luminal tumours [14,15]. Considering that luminal tumours are associated with longest disease-free survival [2], and also that increased nuclear localization of PAX2 in tumour cells negatively correlates with tumour recurrence [15], preferential activation of PAX2 in poorly metastatic luminal breast cancer cells suggests that PAX2 activity confers protection against metastasis in these tumours. Supporting this hypothesis, a recent study has revealed that PAX2 negatively regulates the expression of a well-established pro-invasion and pro-metastastic gene, ERBB2 [5,6], in estradiol-treated luminal breast cancer cell lines [17]. A putative role for PAX2 as a negative regulator of invasion and metastasis in breast cancer cells, however, has not been pursued to this day.

In the present study, we have characterized the regulation of PAX2 expression and activation in luminal and non-luminal breast cancer cells; we also examined how the modulation of PAX2 expression affects the invasiveness of estradiol-treated luminal breast cancer cells. Our results show that in response to estradiol, PAX2 is phosphorylated and activated in breast cancer cells of the luminal subtype selectively, via ERα; further, modulation of PAX2 expression in these cells directly impacts their invasive ability, in an ERα-dependent manner.

## Results

**Figure 1:**
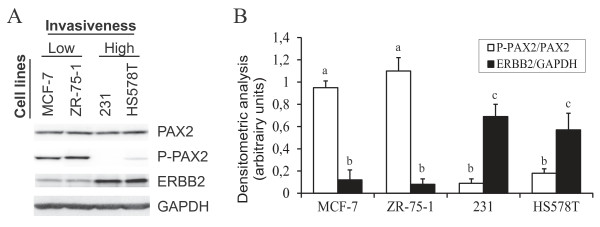
**PAX2 is activated in breast cancer cell lines of the luminal subtype selectively**. Breast cancer cell lines of the luminal (MCF-7 and ZR75-1) and non-luminal (MDA-MB-231 (231) and HS578T) subtypes were cultivated in their standard growth medium, containing growth factors, steroid hormones and estradiol-mimicking molecules such as phenol red. A) Total levels of PAX2 protein, PAX2 phosphorylation on ser393 residue, and ERBB2 protein content were determined using western blot. GAPDH was used as a control to normalize for protein content. Results are representative of three independent experiments. B) Densitometric analysis of the results presented in A. Values are mean +/-SD of three independent experiments. Different subscript letters indicate a statistically significant difference.

To characterize PAX2 regulation in breast cancer cells, we first compared basal expression and activation of PAX2 between luminal and non-luminal cell lines. PAX2 protein levels were similar in all tested cell lines when cultivated in standard medium containing steroid hormones and estradiol-mimicking molecules (Figure 1a), indicating that PAX2 expression does not differ between the two cell subtypes. However, PAX2 phosphorylation on serine 393 residue, a marker of its activation [18], was higher in luminal/ERα+ cell lines (MCF-7 and ZR-75-1 cells [8]) compared to non-luminal/ERα- cell lines (MDA-MB-231 and HS578T [8]) (Figure 1a,b), showing preferential activation of PAX2 in luminal breast cancer cells *in vitro*, as observed *in vivo *[14,15]. The highest levels of phosphorylated PAX2 protein were detected in the least invasive cell lines (luminal cell lines MCF-7 and ZR-75-1 [8], Figure 1b), and negatively correlated with protein levels of ERBB2 (Figure 1b), a major player in breast cancer cell invasion and metastasis [5,6,19]: these results support the hypothesis that PAX2 activation in luminal breast cancer cells maintains a mild invasive and metastatic behavior.

**Figure 2:**
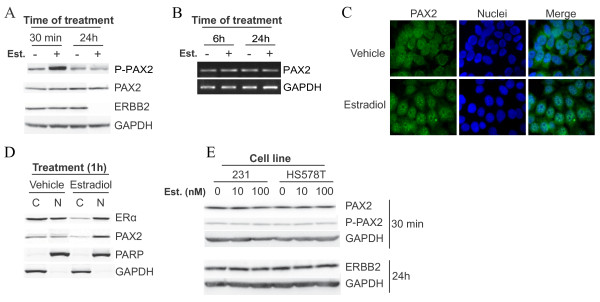
**PAX2 is activated by estradiol in breast cancer cells of the luminal subtype selectively**. Breast cancer cells were cultivated in a steroid- and serum-depleted growth medium, before they were treated with estradiol. A) MCF-7 cells were treated for the indicated time periods with 10 nM estradiol and total levels of PAX2 protein, PAX2 phosphorylation on ser393 residue, and ERBB2 protein levels were determined using western blot. GAPDH was used as a control to normalize for protein content. B) MCF-7 cells were treated for the indicated time periods with 10 nM estradiol and the impact on PAX2 gene expression were determined using RT-PCR. GAPDH was used as a control to normalize for total mRNA content. C-D) MCF-7 cells were treated with 10 nM estradiol for 1 h and subcellular localization of PAX2 was determined using immunofluorescence (C) and subcellular fractionation (D). In C), Hoescht dye was used to visualize cell nuclei; magnification: 400×. In D), ERα was used as a positive control for estradiol-induced nuclear import, PARP was used as a marker of nuclear fraction (N) purity, and GAPDH was used as a marker of cytosolic fraction (C) purity. E) Non-luminal breast cancer cell lines MDA-MB-231 (231) and HS578T were treated with the indicated doses of estradiol. Following 30 min-treatment with estradiol, PAX2 phosphorylation on ser393 residue and total levels of PAX2 protein were determined, whereas following 24 h-treatment with estradiol, total levels of ERBB2 protein were determined, using western blot. GAPDH was used as a control to normalize for protein content. All results are representative of three independent experiments.

We have then determined the impact of estradiol exposure on the activation status of PAX2, in luminal and non-luminal breast cancer cell lines. To this aim, breast cancer cells were cultivated in a steroid- and serum-depleted growth medium, before they were treated with exogenous estradiol at concentrations known to activate estrogen-responsive genes in luminal breast cancer cell lines such as MCF-7 cells (100 nM or less) [20]. A 30-min treatment with estradiol increased the levels of phosphorylation of PAX2 protein on serine 393 residue in MCF-7 cells (Figure 2a), and induced the accumulation of PAX2 protein in cell nucleus, as shown by immunofluorescence (Figure 2c) and subcellular fractionation (Figure 2d). In addition, a longer treatment with estradiol (24 hours) led to a reduction of ERBB2 protein levels in MCF-7 cells (Figure 2a), in agreement with a previously described role for PAX2 in the regulation of ERBB2 gene expression in these cells upon treatment with estradiol [17]. Since estradiol treatment had no impact on PAX2 expression, at the protein (Figure 2a) or mRNA (Figure 2b) levels, these results indicate that estradiol specifically regulates the activation of PAX2 protein, but not PAX2 expression, in luminal breast cancer cells. Treatment of non-luminal cell lines HS578T and MDA-MB-231 with exogenous estradiol had no impact on PAX2 phosphorylation and ERBB2 protein content (Figure 2e), showing that estradiol does not regulate PAX2 activity in these cells. Collectively, these results indicate that PAX2 activation in response to estradiol is selectively achieved in breast cancer cells of the luminal subtype.

**Figure 3:**
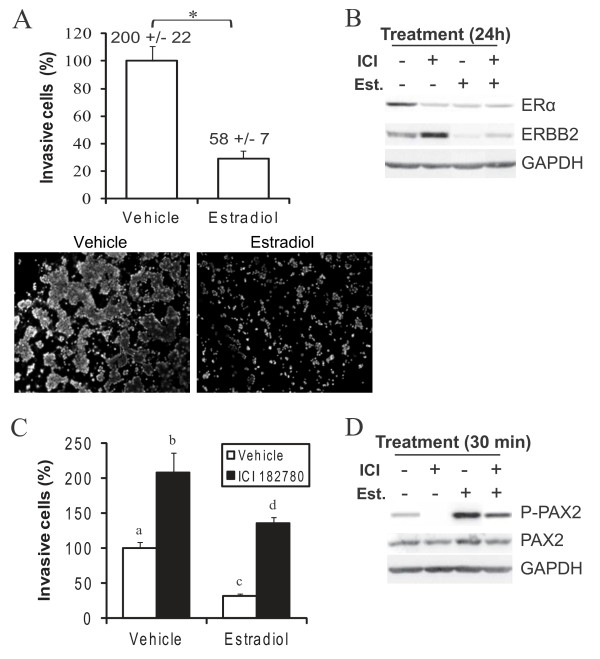
**Estradiol negatively regulates PAX2 activity and invasion in an ERα-dependent manner in MCF-7 cells**. A) MCF-7 cells were cultivated in a steroid- and serum-depleted growth medium and treated with 10 nM estradiol for 24 h before they were subjected to Matrigel invasion assay. Numbers above bars indicate total number of cells recovered in the lower chamber at the end of the assays. Pictures show the density of invasive cells that reached the porous filter. B-D) MCF-7 cells were cultivated in a steroid- and serum-depleted growth medium and pretreated with ERα antagonist ICI 182780 (1 μM) for 1 h before they were treated with 10 nM estradiol. (B) Following 24 h-treatment with estradiol, total protein content of ERBB2, and of ERα as a marker of ICI 182780 efficiency, were determined using western blot. GAPDH was used as a control to normalize for protein content. (C) Following 24 h-treatment with estradiol, cell invasion was determined using Matrigel invasion assay. (D) Following 30 min-treatment with estradiol, the extent of phosphorylation of PAX2 on ser393 residue and total levels of PAX2 protein were determined, using western blot. GAPDH was used as a control to normalize for protein content. All results are representative or mean value +/-SD of three independent experiments. Different subscript letters and * indicate a statistically significant difference.

By downregulating ERBB2 expression in luminal breast cancer cells [17,21], estradiol could negatively regulate their invasiveness; this question, however, had not been directly examined to this day. We found that overall invasion of MCF-7 cells was indeed reduced by estradiol (Figure 3a). ICI 187280 (ICI) efficiently antagonizes ERα in these cells, as shown by long-term downregulation of ERα expression (Figure 3b) [22,23]; in these conditions, MCF-7 cell invasion was increased (Figure 3c), PAX2 phosphorylation was reduced (Figure 3d) and ERBB2 protein levels were increased (Figure 3b), whether the cells were subsequently treated with estradiol or not. Collectively, these results demonstrate a role for ERα in basal and estradiol-induced PAX2 activation, ERBB2 downregulation and decrease of cell invasion in luminal MCF-7 cells.

**Figure 4:**
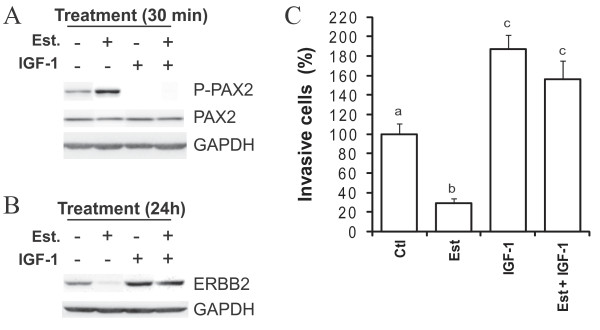
**IGF-1 antagonizes estradiol-induced activation of PAX2 in luminal breast cancer cells**. MCF-7 cells were cultivated in a steroid- and serum-depleted growth medium and treated with 10 nM estradiol and/or 30 ng/mL IGF-1, as indicated. A) Following 30 min-treatment with estradiol and/or IGF-1, PAX2 phosphorylation on ser393 residue and total levels of PAX2 protein were determined using western blot. GAPDH was used as a control to normalize for protein content. B) Following 24 h-treatment with estradiol and/or IGF-1, total levels of ERBB2 protein were assessed, using western blot. GAPDH was used as a control to normalize for protein content. C) Following 24 h-treatment with estradiol and/or IGF-1, cells were subjected to Matrigel invasion assay. All results are representative or mean values +/- SD of three independent experiments. Different subscript letters indicate a statistically significant difference.

To further document a relationship between PAX2 activity and cell invasion, we have examined whether insulin-like growth factor-1 (IGF-1), which positively regulates ERBB2 expression in luminal cells [24] and increases the motility and invasiveness of MCF-7 cells [25,26], regulated PAX2 activation by estradiol in these cells. Treatment of cells with IGF-1 led to a rapid reduction of PAX2 phosphorylation (Figure 4a), contrary to estradiol which increased P-PAX2 levels (Figure 4a). When IGF-1 and estradiol were combined, PAX2 phosphorylation was reduced (Figure 4a), indicating that IGF-1 antagonizes the effect of estradiol on PAX2 phosphorylation. In agreement, treatment of MCF-7 cells with IGF-1, alone or in combination with estradiol, increased ERBB2 protein levels (Figure 4b), as well as cell invasion (Figure 4c). Altogether, these results strengthen the inverse relationship between PAX2 activity and cellular invasion and identify IGF-1 as a negative regulator of PAX2 activity in luminal breast cancer cells.

**Figure 5:**
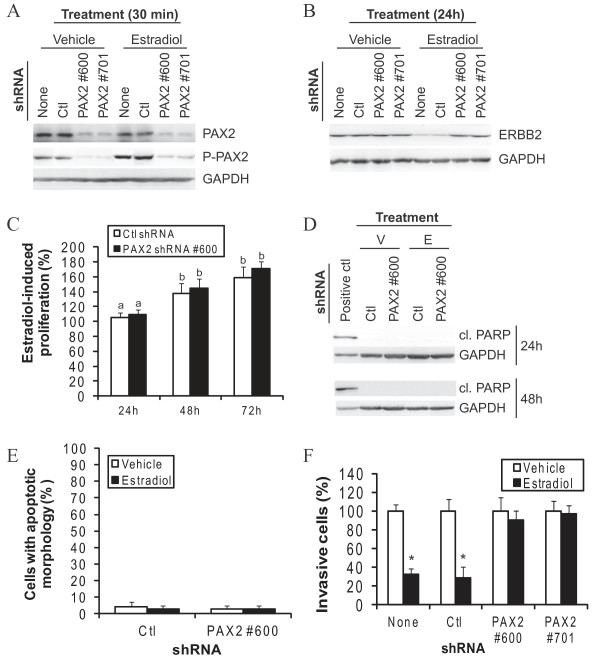
**Knockdown of PAX2 abrogates estradiol-induced decrease of cell invasion in luminal breast cancer cells**. MCF-7 cells were cultivated in a steroid- and serum-depleted growth medium, transfected with PAX2 shRNAs, control shRNA (Ctl: scrambled sequence from PAX2 shRNA #600) or transfection reagent only (None) for 24 h and then treated with 10 nM estradiol. A) PAX2 phosphorylation on ser393 residue and total levels of PAX2 protein were determined using western blot after 30 min of treatment with estradiol. B) Total levels of ERBB2 was determined following 24 h-treatment with estradiol. GAPDH was used as a control to normalize for protein content. C-D) Following treatment with estradiol for the indicated times, cell proliferation was determined, using MTT assay (C), and the presence of cleaved fragments of PARP was assessed using western blot (D). Positive control for PARP cleavage was MCF-7 cells treated with 10 μM cisplatin for 24 h. E-F) Following 24 h-treatment with estradiol, cell nuclei were stained with Hoechst dye and number of apoptotic cells was determined under a fluorescent microscope (E), and cells were subjected to Matrigel invasion assay (F). All results are representative or mean value +/-SD of three independent experiments; different subscript letters indicate a statistically significant difference.

To directly address a putative role for PAX2 in estradiol-induced decrease of cell invasion, we knocked down PAX2 using two different small hairpin RNAs (shRNAs). Both shRNAs efficiently decreased total and phosphorylated PAX2 protein levels in MCF-7 cells (Figure 5a), and completely prevented estradiol from decreasing ERBB2 expression (Figure 5b). They had no impact on estradiol-induced cellular proliferation (Figure 5c), and did not induce cleaved fragments of PARP, a marker of apoptosis (Figure 5d), or increase the number of cells with apoptotic nuclear morphology (Figure 5e). PAX2 knockdown therefore did not impact cellular proliferation and viability; however, it completely abrogated estradiol-induced decrease of cell invasion (Figure 5F). These results reveal for the first time a role for PAX2 in the regulation of cell invasion.

**Figure 6:**
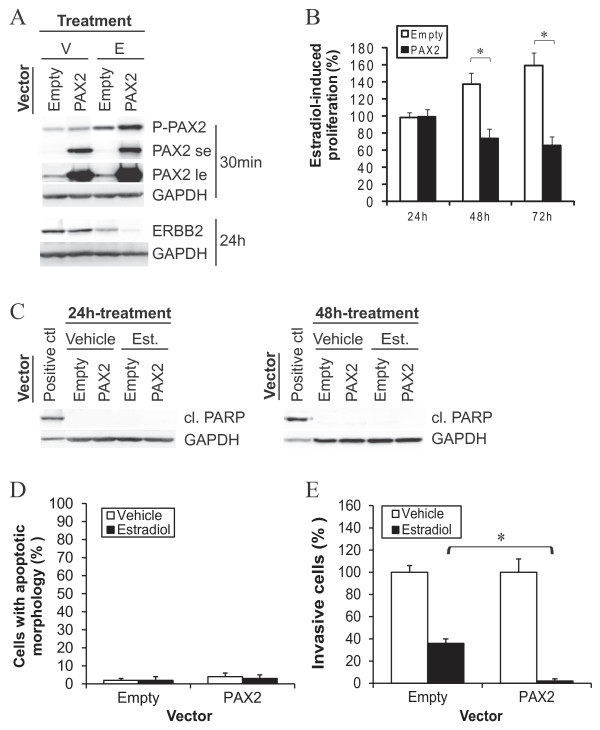
**Overexpression of PAX2 enhances estradiol-induced decrease of cell invasion in luminal breast cancer cells**. MCF-7 cells were cultivated in a steroid- and serum-depleted growth medium, transfected with PAX2 cDNA or empty vector and then treated with 10 nM estradiol. A) Following 30 min-treatment with estradiol, PAX2 phosphorylation on ser393 residue and total levels of PAX2 protein were determined (**se**: short exposition time, to ensure comparable levels of exogenous PAX2 protein between PAX2-transfected cells treated with estradiol or not; **le**: long exposure time, to detect endogenous PAX2 protein in empty vector-transfected cells), whereas following 24 h-treatment with estradiol, total levels of ERBB2 protein were determined, using western blot. GAPDH was used as a control to normalize for protein content. Results are representative of three independent experiments. B) Following treatment with estradiol for the indicated times, cell proliferation was determined, using MTT assay. Results are mean +/- SD of four independent experiments. *statistically significant difference. C) Following treatment with estradiol for the indicated times, the presence of cleaved fragments of PARP was assessed using western blot. Positive control for PARP cleavage was MCF-7 cells treated with 10 μM cisplatin for 24 h. D) Following 24 h-treatment with estradiol, cell nuclei were stained with Hoechst dye and number of apoptotic cells was determined under a fluorescent microscope. E) Following 24 h-treatment with estradiol, cells were subjected to Matrigel invasion assay. In C-E, results are representative or mean value +/-SD of three independent experiments. *statistically significant difference.

Finally, we determined if overexpression of PAX2 could successfully decrease the invasive ability of luminal cells. Transfection of MCF-7 cells with a plasmid vector containing PAX2 cDNA increased total levels of PAX2 protein (Figure 6a): the exogenous protein was phosphorylated in response to estradiol and ERBB2 protein content was efficiently decreased (Figure 6a). Overexpression of PAX2 inhibited estradiol-induced cellular proliferation (Figure 6b), but did not induce the cleavage of PARP (Figure 6c) or increase the number of cells with apoptotic nuclear morphology (Figure 6d). It enhanced, on the other hand, estradiol-induced decrease of ERBB2 protein content (Figure 6a) and cell invasion (Figure 6e), showing that specific delivery of PAX in luminal cells exposed to estradiol efficiently reduces their invasive ability.

## Discussion

Multiple factors influence the metastatic outcome of a cancer, many of them extending beyond intrinsic tumour cell properties. Nonetheless, it is now recognized that as a group, luminal primary breast tumours have a distinct molecular profile and display weak invasive and metastatic behavior [2,3,27,28]. Identifying key molecules and mechanisms, which prevent luminal tumours cells from evolving towards an aggressive metastatic phenotype, should foster the development of new anti-metastatic strategies.

The presence of PAX2 in breast tumours has been described many years ago [14,16], but the role of the transcription factor in these tumours remained unknown until recently, when PAX2 was shown to dictate the response of luminal breast cancer cells to tamoxifen [17]. In the present study, we describe a new role for PAX2 in luminal breast cancer cells, as a negative regulator of cell invasiveness. Because the majority of breast cancer patients bear luminal tumours [29], these new informations have important clinical relevance. Further, we provide proof-of-concept for the efficiency of PAX2 overexpression to decrease cell invasion. We observed that PAX2 overexpression also prevented estradiol from inducing the proliferation of luminal breast cancer cells and therefore, apart from interfering with the metastatic progression of the disease, therapeutic overexpression of PAX2 could induce cytostatic effects in pre-existing primary and metastatic breast tumours. Available clinical data indeed suggests an inverse relationship between PAX2 activity and the proliferation of breast cancer cells *in vivo*: lower PAX2 immunoreactivity can be detected in the nucleus of cells from high grade breast tumours compared to low grade tumours [14].

Considering such a key role for PAX2 in the control of luminal breast cancer cell invasiveness, understanding the mechanisms regulating its activity is primordial. We show here that PAX2 protein is activated by estradiol, in breast cancer cells of the luminal subtype only, in agreement with clinical data showing preferential activation of PAX2 in tumours of the luminal subgroup *in vivo *[14,15]. ERα is required for estradiol to induce PAX2 activation; it is also required for estradiol to decrease ERBB2 expression and cellular invasion, in agreement with previous studies showing that antagonizing ERα increases the invasiveness of MCF-7 cells [30]. It is worth noting that in luminal breast cancer cells, exposure to estradiol has no impact on total PAX2 mRNA and protein levels; in addition, total levels of PAX2 protein are comparable in luminal versus non-luminal breast cancer cells *in vitro*, similar to clinical data showing the presence of PAX2 in luminal as well as non-luminal tumours *in vivo *[14,15]. Therefore, transcription would not be a major level of regulation for PAX2 in breast cancer cells, compared to post-translational modification of the protein. Finally, we have identified a first negative regulator of PAX2 activity in luminal breast cancer cells, IGF-1. The inhibition of anti-invasive factors such as PAX2 may contribute to the pro-invasive and pro-metastatic effects of IGF-1 in breast cancer cells [25].

Results from previous reports indirectly suggested that exposure to estradiol negatively regulates breast cancer cell invasion: overexpression of ERα in non-luminal breast cancer cells decrease their invasive ability [31] and conversely, exposure to anti-oestrogens such as tamoxifen can increase the invasive ability of ERα+ breast cancer cells [32]. Indeed, although the main effect attributed to tamoxifen is growth inhibition, it has also been shown to induce an invasive phenotype in breast cancer cells, notably by inducing the expression of matrix metalloproteinases as described in cultivated MCF-7 cells [33]. The results from the present study now directly demonstrate that exposure to estradiol has an opposite effect to tamoxifen and inhibits the invasion of breast cancer cells, at least for those of the luminal subtype. It may appear contradictory that estradiol, which initially promotes the development of luminal (estrogen-responsive) breast tumours by increasing the proliferation of breast epithelial cells [34,35], would later repress the invasion and metastasis of established luminal tumour cells. However, available clinical data supports this paradigm. It was reported, for example, that women using hormone replacement therapy (HRT) had an increased risk of developing breast cancer, but those who did develop a breast cancer presented with more localized tumours and had a more favorable prognosis than women who did not use HRT [32]. Also, in patients with ERα+ breast tumours presenting with metastasis, administration of estradiol pills induced clinical benefits: most patients had longer progression free survival [36]. In the light of the present work, it is likely that PAX2 activation and downregulation of ERBB2 expression directly contributes to these beneficial effects of estradiol.

## Conclusion

Identifying PAX2 as a natural barrier against invasion (and possibly, metastasis) in ERα+/luminal breast cancer cells, and showing that overexpression of PAX2, when it is followed by its activation, is sufficient to reduce cellular invasiveness of luminal breast cancer cells, is of clinical relevance considering that the majority of patients with breast cancer present with a luminal tumour and that metastasis is the main cause of death in breast cancer patients. It also has the potential to be extended to non-luminal tumours, even though estrogen signaling is disrupted in ERα- tumour cells. Indeed, the mechanisms underlying ERα downregulation in non-luminal breast cancer cells have already been scrutinized and several promising strategies to restore ERα expression are currently under evaluation (reviewed in [36,37]). Therefore, we can realistically envision future combination therapies, designed to re-express ERα and to overexpress PAX2 at the same time in non-luminal breast cancer cells, capable of efficiently interfering with progression of the disease towards metastasis.

## Methods

### Cell lines and reagents

Human breast carcinoma cell lines (ERα-positive MCF-7 and ZR-75-1 cells and ERα-negative MDA-MB-231 and HS578T cells) were generously provided by Dr Eric Asselin (University of Quebec in Trois-Rivières, QC, Canada), who had initially purchased the cell lines from ATCC. All cell lines were routinely maintained in RPMI-1640 medium containing 50 μg/ml gentamycin, and supplemented with bovine growth serum (10% for MCF-7 cells and 5% for MDA-MB-231 and HS578T cells) or fetal bovine serum (10% for ZR-75-1 cells). All antibodies were from Cell Signaling Technology (Beverly, MA, USA) except for HRP-conjugated goat anti-rabbit secondary antibody (Bio-Rad Laboratories, Mississauga, ONT, Canada), ERα (Ab-16 from Neomarkers, Thermo Fisher Scientific, Fremont, CA, USA), PAX2 (sc-130387 from Santa Cruz Biotechnology, Santa Cruz, CA, USA) and phospho-PAX2 (71-6000 from Invitrogen, Burlington, ONT, Canada) antibodies. Estradiol and recombinant human IGF-1 were purchased from Sigma-Aldrich (Oakville, ONT, Canada) and ERα antagonist ICI 182780 was purchased from Cedarlane Laboratories (Burlington, ONT, Canada).

### Cell treatments

Before treatments with estradiol, cells were progressively adapted to medium depleted of growth factors, steroid hormones, and estradiol-mimicking molecules such as phenol red. Specifically, cells were seeded in 6-well plates (1 × 10^6 ^cells per well for MCF-7 and HS578T cells, and 3 × 10^6 ^cells per well for ZR-75-1 and MDA-MB-231 cells) and allowed to adhere and proliferate in standard medium containing serum for 72 h. They were washed twice with PBS and incubated for 24 h in RPMI-1640 without phenol red containing 2% dextran-charcoal treated fetal bovine serum. Finally, cells were washed and incubated for 24 h in RPMI-1640 without phenol red or serum. Then, cells were treated with estradiol (10 nM or as indicated), or with vehicle (dimethylformamide), in RPMI-1640 without phenol red. When needed, they were pre-treated with 1 μM ICI 282 780 or with vehicle (DMSO) for 1 h before estradiol treatment. In all cases, adherent and floating cells were collected at the end of the treatment.

### Western blots analysis

Treated cells were disrupted in cold RIPA buffer containing protease inhibitors (Complete™ from Roche, Laval, Quebec, Canada), followed by three freeze-thaw cycles. Equal amounts of cell lysates were separated onto 10% polyacrylamide gels and then transferred onto nitrocellulose membranes (Bio-Rad). The membranes were probed with primary antibody overnight at 4°C and incubated with horseradish peroxidase-conjugated secondary antibody for 45 min. Detection was performed using SuperSignal West Femto substrate (Pierce, Arlington Heights, IL, USA), as described by the manufacturer.

### Immunofluorescence analysis

Cells were grown on glass coverslips before they were treated as described above. Treated cells were fixed with 4% paraformaldehyde for 10 min, washed twice in phosphate buffered saline (PBS) and permeabilized for 10 min in citrate solution (0.1% sodium citrate, 0.1% Triton X-100 in PBS) at room temperature. They were incubated at room temperature for 1 h with primary antibody (PAX2, #71-6000 from Invitrogen, diluted 1/20 in PBS), then for 30 min at room temperature and in the dark with secondary antibody (Alexa Fluor 488-conjugated donkey anti-rabbit IgG antibody, A-21206 from Invitrogen, diluted 1/200 in PBS). Cell nuclei were counterstained with 0.25 mg/mL Hoechst dye (Sigma) for 3 min, before coverslips were added onto cells. Stained cells were visualized under a fluorescence microscope.

### RT-PCR analysis of PAX2 expression

Total RNA was isolated from treated MCF-7 cells using Trizol Reagent (Invitrogen, Burlington, ON, Canada) according to manufacturer's instructions. First strand cDNA was synthesized from 0.4 μg RNA using MMLV reverse transcriptase (Invitrogen). Primers for PCR amplification of PAX2 were 5'- GTACTACGAGACCGGCAGCATC-3' (sense) and 5'- CGTTTCCTCTTCTCACCATTGG-3' (antisense) and primers for amplification of GAPDH were 5'-gtcagtggtggacctgacct-3' (sense) and 5'-tgagcttgacaaagtggtcg-3' (antisense). PCR reactions were conducted in a MJ Research Thermal cycler (model PTC-100), using the following parameters: 30 sec. at 94°C, 30 sec. at 58°C, and 1 min. at 72°C, for 35 cycles (PAX2) or 25 cycles (GAPDH). The reaction mixtures were size-separated on an agarose gel and visualized using SYBR-SafeTM (Invitrogen) staining upon ultraviolet transillumination.

### Subcellular fractionation

NE-PER Nuclear and Cytoplasmic Extraction Reagent (Thermo Fisher Scientific) was used according to the manufacturer's instructions.

### Matrigel invasion assay

The invasive properties of treated cells were measured using Matrigel-coated Transwell inserts (Costar, Corning, NY, USA). Briefly, inserts with 8-μm pore size were coated with 2 mg/ml Matrigel (VWR, Mississauga, ON, Canada), and treated cells were collected, washed, and resuspended in respective basal medium without serum or phenol red. The lower chambers were filled with 600 μl of respective culture medium, and 2 × 10^5 ^cells were added to the upper chamber inserts. The plates were incubated for 72 h at 37°C. After this incubation period, invasive cells had crossed the porous barrier and reached the lower compartment (as previously reported by others [38,39]). Medium filling the lower chamber was collected, and invasive cells were recovered by centrifugation (1 min at 6,000 × g) before they were resuspended in 30 μl of PBS and counted using a hemocytometer. A percentage of cell invasion was calculated from the ratio of total number of cells recovered from the lower compartment to the total number of cells initially loaded in the upper compartment. To obtain the pictures presented in A, Matrigel was removed/wiped away from the filters after 24 h incubation, and cells having reached the filters at this time point were fixed with cold methanol and stained with Hoechst 33258 dye (Sigma). Pictures were taken under a fluorescence microscope (magnification: 100×).

### Transfection with vectors encoding PAX2 shRNAs or cDNA

MCF-7 cells were seeded in 6-well plates at the required density to reach approximately 60% confluency after 24 h. The day of transfection, PAX2 shRNA constructs or PAX2 cDNA constructs were added to cells using a ratio of 3.6 μL Fugene:1.2 μg DNA/well. Transfection reagent alone was also separately added as a control treatment, using the same dilution as described above. After 8 h-transfection, medium was replaced and plates were incubated for 40 additional hours (total: 48 h) at 37°C before cells were used for subsequent treatment with estradiol. PAX2 shRNA constructs were: 5'- GAGGAAACGTGATGAAGAT-3' for PAX2 shRNA #600; 5'-CCCAGCAGCAGCTGGAAGC-3' for PAX2 shRNA #701; 5'-GCAATGACGCAGGTGACCA-3' for control (Ctl) shRNA (scrambled sequence from PAX2 shRNA #600). All shRNAs were inserted into pSilencer 1.0-U6 vector; the constructs were a kind gift from Dr Yoshiko Takahashi, Nara Institute of Science and Technology, Nara, JAPAN. The PAX2 cDNA constructs were: human PAX2 cDNA in pcDNA3.1 vector, or empty vector as a control. The constructs were a kind gift from Dr Paul Goodyer, McGill University, Montréal, Qc, CANADA.

### MTT proliferation assay

Cells were transfected with PAX2 cDNA or shRNA constructs as described above, with the exception that the incubation following 8-h transfection period was 24 h instead of 48 h. They were then trypsinized, counted and plated in 96-well plates at a density of 1 × 10^4 ^cells per well, and incubated for an additional 24 h-period at 37°C, after which they reached 80% confluence. Estradiol was added to selected wells at the concentration of 10 nM in 100 μl culture medium (total time between initial transfection and estradiol treatment: 48 h), and plates were incubated for indicated times at 37°C. MTT reagent (3-(4,5-Dimethyl-2-thiazolyl), Sigma) was added to the wells (10 μl of a 0.5% solution in PBS) 3.5 h before the end of the incubation period, and conversion of yellow tetrazolium salt to blue thiazol crystals by metabolically active cells was stopped by adding 100 μl of a 10% sodium dodecyl sulfate, 0.1% HCl solution to each well. Plates were incubated overnight at 37°C to allow complete solubilization of thiazol crystals, and intensity of blue emission in each well was measured using FluoStar multiwell plate reader (BMG Laboratories, Durham, NC). Percentage of proliferating cells was calculated as the ratio of optical densities of estradiol-treated to control-treated cells.

### Determination of apoptosis using Hoechst nuclear staining

Treated cells were collected, washed twice in PBS, resuspended at an approximate density of 2 × 10^5 ^cells/ml in PBS containing 1 μg/ml Hoechst 33258 (Sigma) and 2% formalin and incubated for 24 h at 4 °C, before blind cell counts of apoptotic cells was carried out under a fluorescence microscope. At least 200 cells were counted for each sample, and a percentage of apoptotic cells was calculated as the ratio of apoptotic cells (with characteristic apoptotic morphology such as nuclear shrinkage and condensation) to total cell count.

### Statistical analyses

Data were subjected to one-way ANOVA (PRISM software version 3.03; GraphPad, San Diego, CA, USA). Differences between cell groups were determined by the Tukey's test. Statistical significance was accepted when P < 0.05.

## List of Abbreviations

231: MDA-MB-231; ER: estrogen receptor; ERα: estrogen receptor alpha; ERBB2: erythroblastic leukemia viral oncogene homolog 2; GPR30: G protein-coupled receptor 30; IGF-1: insulin-like growth factor 1; MAPK: mitogen-activated protein kinase; PARP: poly(ADP-ribose) polymerase; PAX2: paired box 2; PI3-K: phosphatidyl inositol 3-kinase.

## Competing interests

The authors declare that they have no competing interests.

## Authors' contributions

DB and CL carried out the western blot analyses; CVT conducted the invasion, MTT and Hoescht dye staining experiments. DB participated in the statistical analyses and prepared the first draft of the manuscript. CVT conceived the study, participated in its design and coordination and helped to draft the manuscript. All authors read and approved the final manuscript.
